# The Emerging Role of Dietary Bacteriophage in Monogastric Animals in the Post-Antibiotic Era—A Review

**DOI:** 10.3390/vetsci12121146

**Published:** 2025-12-01

**Authors:** Vetriselvi Sampath, Nam Gyun Kim, In Ho Kim

**Affiliations:** 1 Department of Animal Biotechnology, Dankook University, Cheonan 31116, Republic of Korea; suve2314@gmail.com (V.S.); ngkim@ctcbio.com (N.G.K.); 2 Smart Animal Bio Institute, Dankook University, Cheonan 31116, Republic of Korea

**Keywords:** alternative, antibiotics, bacteriophages, pigs, poultry

## Abstract

Bacteriophages, or phages, are viruses that infect and kill specific bacteria, offering a promising alternative to antibiotics in livestock production. With growing concerns over antibiotic resistance, phages have gained renewed interest due to their ability to target harmful pathogens without disturbing beneficial gut microbiota. This review explores the application of dietary phage supplementation in monogastric animals, particularly pigs and poultry. In pigs, phage use has demonstrated beneficial effects such as improved growth performance, enhanced gut health, and reduced infections from *Salmonella* and *E. coli*. Various delivery methods, including feed and water supplementation, have been studied, with microencapsulation showing promising results for stability and effectiveness. Similarly, in poultry, phages have been successfully used to control pathogens like *Salmonella*, *Campylobacter*, and avian pathogenic *E. coli*, improving gut health, immunity, and overall performance. Several commercial phage products are already in use, demonstrating both safety and efficacy. Despite these advantages, challenges such as a narrow host range, bacterial resistance, and regulatory limitations remain. Therefore, further research is necessary to understand phage–host interactions, optimize delivery strategies, and evaluate long-term effects under normal and disease-free conditions. This review highlights the potential of bacteriophages as safe, targeted, and sustainable alternatives to antibiotics in monogastric animal production, contributing to improved animal health and reduced antibiotic use.

## Simple Summary

Bacteriophages are viruses that selectively infect and lyse bacterial cells. In monogastric animal production systems, including poultry and swine, they are increasingly recognized as a viable alternative to conventional antibiotics. Owing to their high host specificity, phages can effectively target pathogenic bacteria such as *Salmonella* spp. and *Clostridium* spp. while largely preserving commensal gut microbiota. This specificity contributes to their safety and environmental compatibility as antimicrobial agents. Recent studies have demonstrated that the inclusion of bacteriophages in feed or water can enhance growth performance, improve gut integrity, and increase nutrient digestibility in both pigs and poultry. By reducing the load of enteric pathogens, phages support a more stable and functionally efficient gut microbial ecosystem, which may contribute to improved digestive processes and immune responsiveness. Unlike antibiotics, bacteriophages do not leave harmful residues in animal tissues or the environment, as they are natural biological entities that degrade after completing their activity. Indeed, effectiveness of phage can be influenced by several factors including type, animal age, diet composition, and storage conditions. Therefore, extensive research is needed to optimize phage formulations to improve animal health and productivity in monogastric production systems.

## 1. Introduction

Bacteriophages, commonly known as phages, are viruses that infect and replicate specifically inside bacterial cells. These intracellular parasites depend on their bacterial hosts for reproduction and are considered the most abundant biological entities on Earth, with their global numbers estimated between 10^31^ and 10^32^ fragments [1]. Phages infect bacteria primarily through two cycles: the lytic and lysogenic cycles. In the lytic cycle, phages rapidly multiply within the host, causing them to burst and release new viral particles that can infect other bacteria. Conversely, during the lysogenic cycle, phage DNA integrates into the bacterial genome, existing as a prophage and replicating alongside the host cell without destroying it. Environmental factors can trigger prophages to switch back into the lytic cycle, producing infectious phage particles. After World War II, the widespread use of antibiotics caused phage therapy to lose momentum and become mostly limited to research. However, recent increases in multidrug-resistant bacteria, largely driven by antibiotic overuse in agriculture and food production, have renewed global interest in phage applications [2]. Phages, also known as living drugs, serve as low-cost antimicrobials [3] and play a vital role in microbial ecology by mediating horizontal gene transfer and influencing bacterial evolution [4]. Their contributions to molecular biology and biotechnology are profound and assist various technologies. Recently, their utility has extended beyond therapy and has been successfully used for bio-decontamination in agricultural applications including swine and poultry production [5]. Given the urgent need for antibiotic alternatives and the promising applications of phages across in agricultural sectors, research into dietary phage supplementation in monogastric animals especially on pigs and poultry is expanding rapidly. Although majority of this research has involved monogastric animals, this manuscript presents a systematic review on exploring the potential of bacteriophages as precise, residue-free, and sustainable alternatives for enhancing animal health, modulating gut microbiota, and improving productivity in monogastric animals. Also, we believe that this review will provide a novel perspective for future studies on the application of phages in the livestock industry.

## 2. Overview of Phage: History, Structure, and Life Cycle

A comprehensive search of primary, peer-reviewed literature evaluating the effectiveness of bacteriophage applications in reducing or preventing bacterial colonization in monogastric animals were conducted in 2023, using PubMed, Sci-Hub, and Google Scholar search engines. The following terms “broiler + phage, phage + weaning pig, phage+ chicks, and bacteriophage + pigs” were used to find the articles. Retrieved articles were excluded, while original research and review articles which examine the effect of phage in both pigs and poultry were utilized. References within these articles were also examined to identify any additional studies meeting the selection criteria.

Phages are prokaryotic viruses that infect pathogenic bacteria, such as *Clostridium perfringens* (*C. perfringens*), *Campylobacter jejuni*, *Salmonella* spp., *Listeria monocytogenes*, *E. coli*, and *Staphylococcus aureus*, while remaining completely harmless to humans, animals, and plants [6]. The term “phage” originates from the Greek word φαγεῖν (phagein), meaning “to devour,” reflecting their ability to replicate inside bacteria and ultimately kill them [7,8]. Unlike traditional predators that feed on their prey, phages rely on living bacterial hosts for replication and cannot utilize nutrients from dead cells. This principle is often likened to the saying, “The enemy of my enemy is my friend,” [9] as phages naturally target harmful bacteria that affect animal health and productivity. Such phages were first identified by Félix d’Herelle in 1917 [10], who later established the International Bacteriophage Institute in Tbilisi, Georgia. Earlier observations by Hankin had already hinted at the antibacterial activity of phage-containing water. Although phage therapy declined from the 1940s onward due to the rise of antibiotics, the global spread of antibiotic resistance has renewed scientific and commercial interest in phages as sustainable antimicrobial agents [11], particularly because they leave no residues and do not contribute to antibiotic overuse concerns [12]. Furthermore, they are ubiquitous in nature and can be found in diverse environments such as soil, water, and various food products [13], generally measuring between 20 and 200 nanometers.

**Figure 1:**
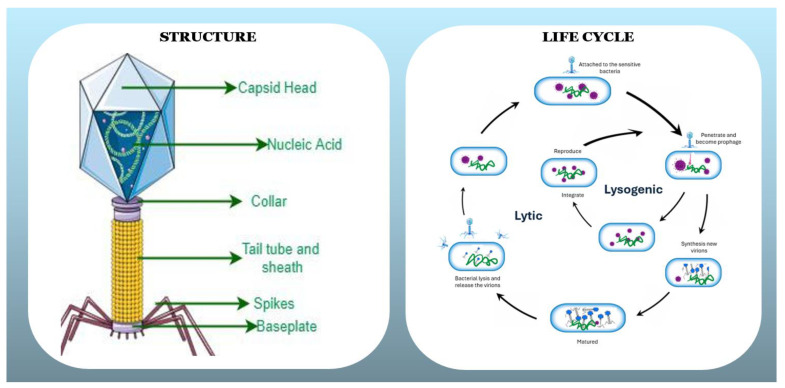
Bacteriophage Structure and Life Cycle: Lytic and Lysogenic Pathways.

Over 5000 phages were examined via electron microscopy. However, their taxonomy has undergone significant changes in recent years. Historically, tailed bacteriophages were classified morphologically under the order *Caudovirales* and the families *Myoviridae*, *Siphoviridae*, and *Podoviridae* [14]. However, phylogenomic studies revealed that these morphology-based groups are paraphyletic and do not accurately reflect evolutionary relationships. Consequently, the International Committee on Taxonomy of Viruses (ICTV) abolished the order *Caudovirales* and the three families, replacing them with a genome-based classification system. Structurally, phages contain either DNA or RNA enclosed in a protein capsid and are classified by genetic material, replication strategy, and host specificity [Figure 1]. Among those 13 families, *Corticoviridae*, *Lipothrixviridae*, *Fuselloviridae*, *Myoviridae*, *Rudiviridae*, *Plasmaviridae*, *Siphoviridae*, *Podoviridae* and *Tectiviridae* represent doubled-stranded DNA, while *Microviridae* and *Inoviridae* were detected as single-strain DNA [15]. To date, host specificity has mainly been determined by their ability to bind the distinct receptors on the bacterial surface. Phages follow two distinct pathways: the lytic cycle and the lysogenic (or temperate) cycle [16], which involves four key stages: adsorption (attachment), infection, multiplication, and release to distinguish replication from bacterial reproduction. Since phages lack their own metabolic systems, they depend entirely on the host bacterium’s cellular machinery for energy production and protein synthesis during replication. In the lytic cycle, they attach to bacterial cells, inject their genetic material, hijack cellular machinery to produce new virions, and ultimately rupture the host cell to release progeny [17]. On the other hand, lysogenic cycle, temperate phages integrate their DNA into the bacterial genome as a prophage, which can persist without killing the host and may carry genes linked to virulence [18,19]. In some cases, a lysogenic phage’s genetic material remains inside the bacterial cell as an independent plasmid rather than integrating into the host chromosome, allowing it to be passed on during cell division [20]. However, environmental stresses such as chemical exposure, UV radiation, or DNA damage can trigger a shift from the lysogenic state to the lytic cycle [21,22]. Given the diversity among bacteriophages, it is essential to identify and select the most suitable phage for each specific application.

## 3. Application of Phage in Monogastric Animal

**Figure 2:**
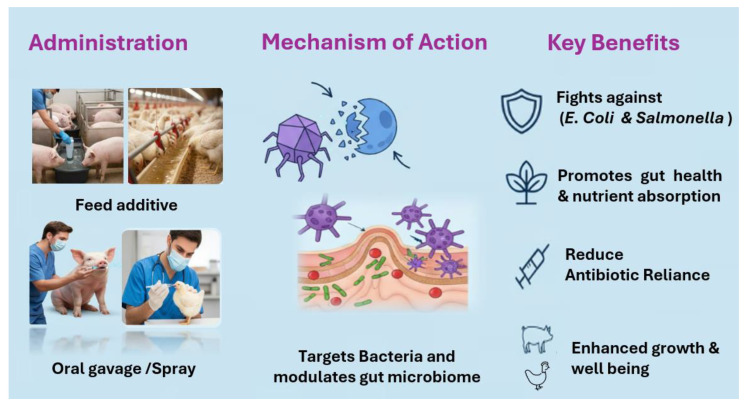
Application and potential effect of phage in monogastric Animal.

The inherent host specificity of viruses offers a promising strategy for combating bacterial infections and decontaminating environments polluted by harmful bacteria. Earlier studies have proven the efficacy of phage therapy in livestock production. For instance, Peh et al. [23] examined the effect of in vitro lytic activity of 18 *Campylobacter*-specific group II phages and 19 group III phages using planktonic killing assay and found a significantly reduced *Campylobacter* counts in cloacal swabs in Ross 308 broilers. Additionally, phages have been found to modulate gut microbiota by promoting beneficial microbial populations and suppressing harmful bacteria, ultimately improving gut health [24] and nutrient utilization in intensive livestock production systems [Figure 2].

This highlights their potential as viable alternatives to in-feed antibiotics. Some studies showed that direct application of phages to the infection site yields the most efficient outcomes [25]. For example, Oliveira et al. [26] conduct an in vivo trail to examine the effect of *Myoviridae* (10^6^ PFU), *Siphoviridae* (10^7^ PFU), and *Myoviridae* (10^8^ PFU) phages in chicks and found that oral administration allowed the phages to remain active in the lungs and support to overcome respiratory *E. coli* infections in broilers. Similarly, a systematic review and meta-analysis by Mosimann et al. [27] concluded that phage delivery through feed has more effective in chickens compared to administration via drinking water or aerosol spray. Phages are particularly sensitive to harsh gastrointestinal conditions, including low pH and digestive enzyme activity, which can limit their viability and therapeutic potential [28]. To address this, Colom et al. [29] investigated a phage cocktail containing three alginate/CaCO_3_-encapsulated phages (UAB_Phi78, UAB_Phi20, and UAB_Phi87) and demonstrated that encapsulation substantially increased both the effectiveness and persistence of the treatment against *Salmonella* infections in poultry, likely by shielding the phages from gastric acidity. It is important to note that the effectiveness of phage therapy is influenced by several critical factors, such as the delivery method (e.g., feed vs. gavage), age of the animal, type of treatment (monophage vs. polyphage), timing of administration (before or after bacterial challenge), duration of treatment, phage concentration [30], formulation, and storage conditions. Moreover, external variables like the phage-to-bacteria ratio, pH, temperature, host accessibility, neutralization, sample collection method, phage isolation technique, and the potential for bacterial resistance must also be carefully considered.

### 3.1. Swine

Pig farming is often conducted under intensive conditions, increasing the risk of bacterial infections that can compromise both health and production performance. These infections not only impair feed efficiency and increase morbidity and mortality but also facilitate the dissemination of antibiotic resistance genes across humans, animals, and the environment, raising broader ecological concerns. Swine health is therefore a critical priority for producers from farrowing to slaughter, as herd infections can reduce weight gain or cause mortality, directly affecting profitability. Diarrheal diseases were most caused by *Escherichia coli*, *Salmonella typhimurium*, and *Clostridium perfringens*, which remain a major challenge in pig production and are associated with substantial economic losses [31]. Previously several studies reported the bacteriophage application in pigs under experimental and dietary conditions [Table 1]. For instance, Saez et al. [32] examined the effect of microencapsulated phage cocktail by gavage at 2, 4, and 6 h after they were challenged with *Salmonella typhimurium* in young pigs and found no significant changes in fecal samples at 6 h post-challenge. However, in the ileum, bacterial levels were significantly lower in phage-treated pigs (1.0 log^10^ CFU/mL) compared with untreated pigs (3.0 log^10^ CFU/mL). Similarly, Albino et al. [33] administered a phage cocktail in finishing pigs and found no significant difference (*p* > 0.05) in the incidence of *Salmonella typhimurium* in fecal samples collected at necropsy. On the other hand, Han et al. [34] evaluated the effect of phage cocktail via feed in weaning pigs challenged with *Escherichia coli* K88 and K99 and found no difference in gut morphology and fecal concentration. Younger pigs are generally more susceptible to infections than older animals, especially to gastrointestinal pathogens. This is also reflected in the datasets we reviewed, which were based on *Salmonella* and *E. coli* challenge models. Unlike water-based, feed-based phage delivery of bacteriophages offers numerous advantages, and recent developments in phage formulation have enabled their conversion into fine powders using excipients through various methods such as lyophilization, freeze-drying, or spray-drying [35]. Previously, Kim et al. [36] reported that supplementing pig diets with commercial phage cocktails targeting *Salmonella*, *E. coli* (K88, K99, F41), *Staphylococcus aureus*, and *Clostridium perfringens* types A and C at inclusion levels of 1000 and 1500 mg/kg improved average daily gain, dry matter digestibility, villus height, and ileal *Lactobacillus* spp. populations, while reducing coliforms and *Clostridium* spp. In a more recent study, the phage EK99P-1 significantly protected intestinal barrier integrity by reducing permeability and preserving tight junction proteins such as zonula occludens-1, occludin, and claudin-3 [37]. In contrast, another study using ileum-inoculated pigs found that dietary antibiotics (0.1%) and phages (0.2%) did not affect ileal or fecal nutrient digestibility compared with the control diet, although both treatments positively influenced the ileal microbiota [38]. The results showed that oral administration of the novel phage cocktail increased body weight gain and improved fecal scores, while reducing neutrophil counts, diarrhea incidence, intestinal inflammation, pathological changes, jejunal loads of SM022 and GN07 bacteria, and the relative abundance of *Enterobacteriaceae*. Additionally, the treatment helped restore gut microbiota composition and diversity [39]. Han et al. [34] reported that adhesion of *E. coli* K88 to piglets showed no significant differences in their ileum and cecal intestinal sections, suggesting that dietary bacteriophages can enhance gut microbiota composition by supporting their intestinal barrier function by reducing inflammation. It is reasonable to hypothesize that the efficacy of phage treatment depends on the bacterial species targeted, since the dataset included only studies using *Salmonella* or *E. coli*, meaning the ability to draw conclusions about phage efficacy against other bacterial groups is limited. Thus, further studies are needed to elucidate the molecular interactions between novel phages, gut microbiota, and host immune response.

### 3.2. Poultry

Chickens are commonly used in phage therapy research. D’Herelle first tested phages in poultry in 1919, curing *Salmonella* with 95–100% success [40]. Unlike antibiotics, phages do not disrupt gut microbiota, supporting beneficial bacteria and reducing secondary infections [41]. This approach is especially important for controlling poultry-associated pathogens such as *C. jejuni*, *Salmonella* spp., *E. coli*, *L. monocytogenes*, and methicillin-resistant *Staphylococcus aureus* (MRSA). Indeed, these pathogens are zoonotic and pose significant threats to human health. Earlier studies have evaluated the efficacy of bacteriophages in reducing bacterial loads and controlling infections in poultry, highlighting their potential as a targeted intervention strategy [42] [Table 2]. For instance, European Food Safety Authority (EFSA) and the European Centre for Disease Prevention and Control (ECDC) identified *campylobacteriosis* as the most frequently reported zoonotic disease in the European Union, followed by *salmonellosis*, Shiga toxin-producing *E. coli* (STEC) infections, and yersiniosis [43]. Such bacteria are widespread in various environments but exhibit a strong preference for colonizing the avian gut, where they typically exist as commensals. In poultry, colonization generally begins around seven days after hatching on farms. Although chickens frequently carry *Campylobacter*, they usually do not show any clinical symptoms or pathological lesions. The prevalence of *Campylobacter* in poultry flocks varies widely, ranging from 2% to 100% depending on the study and sampling conditions [44]. Some studies observed 34.3% *Campylobacter* in cecal samples of broiler chickens [45]. According to a 2018 report by the EFSA and ECDC, the prevalence of *Campylobacter* spp. was 71.6% in turkeys and 26% in broilers. Similarly, farm-level surveillance revealed a significantly higher prevalence of *C. jejuni* (65.8%) compared to *C. coli* (12.6%) [46]. The widespread environmental contamination by *Campylobacter* in slaughterhouses and poultry products is a global concern. Compounding the issue is the growing antibiotic resistance observed in *Campylobacter* strains, particularly against fluoroquinolones, tetracycline, erythromycin, and gentamicin, as well as their increasing virulence, which has spurred efforts to find alternative control measures. Recent experimental studies have demonstrated the application of bacteriophage therapy in reducing *Campylobacter* colonization in poultry, thereby lowering the risk of transmission to humans through contaminated poultry products. In one study, an oral phage cocktail containing lytic *Campylobacter*-specific bacteriophages was administered to broiler chickens colonized with *C. jejuni*. The treatment significantly reduced *C. jejuni* abundance without disrupting the overall gut microbiota. These findings support the use of phage therapy as a targeted intervention to minimize human exposure to *Campylobacter* via the food chain [47]. Despite the promising results, no commercial phage products targeting *Campylobacter* are currently available. This is partly due to the unique challenges associated with *Campylobacter* phages, which differ from other lytic phages in several ways. Difficulties in isolating, propagating, and purifying these phages hinder the development of safe and effective phage cocktails. Additionally, the high cost of production remains a major barrier to commercialization.

*Salmonella* is a major bacterial threat in commercial poultry and the second most significant zoonotic foodborne pathogen after *Campylobacter*. Poultry *Salmonella* infections are mainly classified into host-specific types caused by non-motile serovars, including *Salmonella enterica* serovar Pullorum (*S. pullorum*) and *S. gallinarum*. (1) *S. pullorum* is the causative agent of pullorum disease (PD), a severe systemic infection primarily affecting young chicks, while infected adults typically remain asymptomatic carriers. (2) *S. gallinarum* causes fowl typhoid (FT), a septicemic condition that may occur in acute or chronic forms, mainly affecting growing or adult birds. Non-host-specific infections caused by motile serovars, referred to as paratyphoid (PT) *Salmonellae*, include *S. enteritidis* and *S. Typhimurium*. Additional serovars commonly isolated from poultry include *S. hadar*, *S. infantis*, *S. virchow*, *S. heidelberg*, *S. kentucky*, and *S. anatum*. PT *Salmonella* infections are prevalent but typically do not cause overt disease, except in immunocompromised or stressed young birds (<4 weeks old). These infections are often asymptomatic and characterized by persistent colonization of the intestinal tract and internal organs, which may result in carcass contamination at slaughter. Avian arizonosis (AA), caused by *S. enterica* subsp. *arizonae*, is an acute or chronic infection primarily affecting young turkey poults, with adult birds often serving as asymptomatic carriers.

Consistent with previous study, Berchieri et al. [48] demonstrated the potential of bacteriophages in controlling *Salmonella* colonization in poultry. When *S. typhimurium* and specific bacteriophages were co-administered orally to chickens, notable reductions (>1 log_10_ CFU) in bacterial counts were observed in the crop and small intestine shortly after inoculation and across the gastrointestinal tract by day 3 post-infection. However, some phages (e.g., AB2) exhibited limited efficacy in the cecum. Importantly, no neutralizing antibodies were detected in chicken sera up to 32 days post-inoculation, and phages were recoverable from contact birds, indicating environmental persistence and potential transmission. Subsequent studies confirmed the superior efficacy of phage cocktails over individual phages. For example, a cocktail phage (10^8^ CFU/mL) derived from *S. enteritidis* and *S. typhimurium* ATCC 14028 effectively lysed multiple *Salmonella* serovars, including *S. virchow*, *S. hadar*, and *S. infantis* [49]. Significant reductions in cecal bacterial loads were observed following repeated oral administration of the phage mixture. In one study, no *Salmonella* was detected in the cecum after the fifth dose, indicating effective clearance by day 15 post-infection [50]. Furthermore, the complete genome of a bacteriophage isolated from water using *S. enteritidis* was recently published. This phage demonstrated dual infectivity against both *C. jejuni* and *S. enteritidis*, suggesting potential applications in phage therapy and poultry meat decontamination [51]. Several commercial bacteriophage products targeting *Salmonella* are now available. In 2019, large-scale field trials demonstrated the effectiveness of SalmoFREE^®^, a phage mixture administered via drinking water. The intervention was well-tolerated by broilers, with no negative effects on behavior or performance, and *Salmonella* became undetectable in cloacal swabs by day 33 [52]. Bafasal^®^ (Proteon Pharmaceuticals S.A, Łódź, Poland), administered via drinking water, acts prophylactically and therapeutically, lowering *Salmonella* by up to 200-fold, improving feed conversion, reducing mortality, and requiring no withdrawal period for meat or eggs [53]. Biotector S1^®^ (CJ CheilJedang Research Institute of Biotechnology, Suwon, Republic of Korea) is the first phage-based feed additive developed to target *S. pullorum* and *S. gallinarum*. In a study involving 5-week-old Ross broilers, administration of Biotector S1^®^ at varying doses (5 × 10^7^, 1 × 10^8^, and 2 × 10^8^ PFU/kg feed) resulted in a 73% reduction in mortality compared to the control group (11.81%) following challenge with *Salmonella*. Overall, these findings highlight the growing role of bacteriophages as effective, targeted alternatives to antibiotics for the control of *Salmonella* infections in poultry, with substantial benefits for food safety and animal health.

*Escherichia coli* is a Gram-negative bacillus commonly found in the gastrointestinal tract of birds and is extensively disseminated through feces. Although most *E. coli* strains are nonpathogenic, certain serotypes known as avian pathogenic *E. coli* (APEC) can cause significant disease, leading to increased mortality and carcass condemnations in poultry. These strains act as both primary and secondary pathogens and are prevalent across poultry of all ages. Some APEC strains, such as enterohemorrhagic *E. coli* (EHEC) and its subgroup Shiga toxin-producing *E. coli* (STEC), are foodborne pathogens of major concern to human health globally [54]. Bacteriophages that target *E. coli* are referred to as coliphages. Despite their potential, phage-based commercial products specifically aimed at treating colibacillosis in poultry are not yet widely available. In early work by Barrow et al. [55] bacteriophage R originally isolated from human sewage demonstrated efficacy in preventing and treating septicemia and cerebral infections in chickens. Hu et al. [56] showed that prophylactic administration of aerosolized phages to 7-day-old chicks prevented *E. coli*-induced air sacculitis following a triple bacterial challenge. However, post-infection aerosol treatment was ineffective, likely due to insufficient circulating phage titers. The therapeutic success was therefore dose- and timing-dependent. Furthermore, co-administration of bacteriophages and enrofloxacin resulted in a synergistic improvement in treatment outcomes for colibacillosis. Clavijo et al. [52] evaluated the use of a *Salmonella* phage cocktail (SalmoFREE^®^) in a commercial broiler operation involving 34,680 birds. The phage solution (10^8^ PFU/mL) was administered via drinking water for 2.5 h on days 18, 26, and 34. Compared with control groups, the phage-treated flocks exhibited a significant reduction in *Salmonella* prevalence at slaughter (day 35), without negative impacts on production parameters such as body weight, feed conversion ratio, or mortality. Subsequent analyses indicated that SalmoFREE^®^ did not disrupt the normal development of chicken gut microbiota. In fact, reductions in *Campylobacter* levels and increases in beneficial bacterial genera such as *Butyricimonas*, *Helicobacter*, and *Rikenellaceae* were observed. Similarly, Upadhaya et al. [57] demonstrated that dietary supplementation with phage cocktails (0.05% and 0.1%) targeting *S. gallinarum*, *S. typhimurium*, *S. enteritidis*, *E. coli*, and *Clostridium perfringens* significantly improved broiler growth performance, immune organ development (bursa and spleen), gut microbiota composition, and production metrics. These phage blends were comparable to the antibiotic growth promoter Avilamax^®^ (0.025%), suggesting their potential as effective antibiotic alternatives. Sarrami et al. [58] further reported that broilers receiving the ProBe-Bac phage blend (500–1500 mg/kg) in challenge models involving *Salmonella* and *E. coli* showed enhanced growth, improved intestinal morphology, elevated serum antibody levels, and downregulation of inflammatory markers such as *PGC-1α* and *TLR4* gene expression compared to colistin-treated birds. Encapsulation techniques have emerged as a promising method to improve phage delivery. Encapsulated phages demonstrate improved resistance to gastric acidity, allowing higher viable phage titters to reach the ceca. Lorenzo-Rebenaque et al. [59] showed that encapsulated FGS011 phage, delivered via feed, was more effective at reducing *Salmonella* colonization than non-encapsulated phages. Maciejewska et al. [60] also confirmed that dietary encapsulated phages improved intestinal conditions and reduced *C. perfringens* loads in broiler ceca, compared to powdered phage forms. Combining multiple phages into cocktails is the most widely adopted strategy, as it enhances spectrum coverage and can eliminate diverse *Salmonella* serotypes, improving cross-protection in poultry species, including laying hens [61,62] suggesting that phage therapy is a promising and safe alternative to antibiotics in chicken production as it improves animal health and performance while mitigating zoonotic risks associated with pathogenic bacteria.

**Table 2:** Overview of bacteriophage application in poultry study under experimental and dietary conditions.

Animals	Phage Administration	Target Bacteria	Remarks	Reference
Broilers challenged with *C. jejuni*	Oral—Lytic *Campylobacter*-specific phages	*Campylobacter* spp.	Significant reduction in *C. jejuni* loads; no major disruption of gut microbiota; supports food safety. Commercial phages unavailable due to difficulty in isolating/propagating *Campylobacter* phages.	[45]
Broilers Challenged with *C. jejuni*	Oral—Phage cocktail (multiple lytic phages)	*Campylobacter* spp.	Reduced *C. jejuni* abundance; preserved microbiota composition; shows potential for targeted intervention.	[48]
Chickens	Orally administered with *S. typhimurium*	*Salmonella*- specific phages	>1 log_10_ CFU reduction in crop and small intestine; whole-gut reduction by day 3; no neutralizing antibodies detected; phages recoverable from contact birds.	[49]
Chicks	Orally administered with multi-phage cocktail (10^8^ PFU/mL)	*S. enteritidis* & *S. typhimurium*	Significant cecal *Salmonella* reductions; complete clearance after repeated dosing (day 15).	[51,52]
Broilers	Drinking water—Salmo FREE^®^ phage solution (10^8^ PFU/mL; days 18, 26, 34)	*Campylobacter* spp.	Significant reduction in *Salmonella* at slaughter; no adverse production effects; microbiota stable; secondary reduction in *Campylobacter*.	[53]
Broilers	Drinking water-Bafasal^®^ prophylactic + therapeutic	*Salmonella* spp.	Up to 200-fold reduction in *Salmonella*; improved FCR; reduced mortality; no withdrawal period.	[54]
Chicks	0.5–1.0% of Phage in feed	*S. gallinarum*, *S. typhimurium*, *S. enteritidis*, *E. coli* and *C. perfringens*	Improved growth performance, immune organ indices, microbiota composition; comparable to antibiotic growth promoter (Avilamax^®^).	[58]
Broilers (*Salmonella* + *E. coli* challenge)	ProBe-Bac phage blend (2.04 × 10^8^ PFU/g BP) against *Salmonella* and *E. coli*.		Enhanced growth, better gut morphology, higher antibodies, reduced inflammation (PGC-1α, TLR4 downregulation).	[59]
Broilers	Dietary Encapsulated FGS011 phage	*Salmonella*	Improved survival through GIT; greater *Salmonella* reduction vs. non-encapsulated phages.	[60]

## 4. Bacteriophages: Dual Perspectives on Their Benefits and Limitations

**Figure 3:**
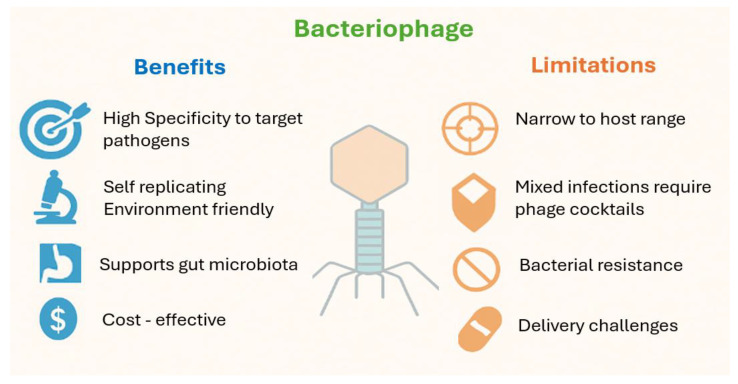
Bacteriophages: Key Strengths and Limitations.

Bacteriophages represent a promising alternative to antibiotics in food animal production, offering several unique advantages and limitations [Figure 3].

One of their most notable attributes is high specificity, as they target bacterial species, serotypes, or even individual strains, thereby minimizing disruption to beneficial microbiota [63]. Unlike broad-spectrum of antibiotics, phages are naturally occurring, self-replicating within host bacteria, environmentally friendly, and cost-effective [63]. Upon administration, phages proliferate at the infection site, providing a self-limiting therapeutic effect and supporting gut microbial balance, reducing the risk of dysbiosis, immune suppression, and secondary infections commonly associated with antibiotic use [64]. They are generally stable at room temperature, resistant to gastric acidity, and maintain viability over time, making them ideal for oral and intestinal delivery [65]. Their production is relatively simple and inexpensive, especially with modern advances in DNA synthesis, high-throughput sequencing, and bioengineering technologies [66]. Beyond their antimicrobial roles, phages have demonstrated potential as immunomodulators and anti-inflammatory agents, broadening their applications in animal health [67]. However, widespread implementation of phage therapy is narrow within the host range, often requiring the use of multi-phage cocktails to combat mixed or diverse infections [68,69]. Effective phage delivery is crucial, as achieving sufficient tiers at the infection site determines therapeutic success. Poor delivery reduces efficacy, highlighting the need for encapsulation or targeted delivery systems [70]. In low- and middle-income countries, challenges include limited clinical data, weak regulatory frameworks, and insufficient scalable manufacturing and quality control [71]. Bacterial resistance to phages is another concern, arising via receptor modification, CRISPR-Cas systems, restriction–modification systems, or abortive infection mechanisms. However, advances in synthetic biology and genetic engineering offer solutions, such as multi-phage cocktails and engineered phages designed to bypass bacterial defenses [72,73,74]. For instance, Thanki et al. [75] identified a three-phage cocktail (CPLM2, CPLM15, CPLS41) that significantly reduced *Clostridium perfringens* colonization in poultry larvae. While phages show strong therapeutic potential, a deeper understanding of their mechanisms and host interactions is needed. Addressing regulatory, production, and commercialization challenges is essential to establish phages as sustainable and effective alternatives to antibiotics in livestock.

## 5. Conclusions and Prospects

Bacteriophages are viruses that are widely distributed in nature and closely associated with their bacterial hosts. Since their discovery, they have been used in various applications, particularly for their strong potential to combat antibiotic resistance. However, standardized guidelines for phage administration and dosage are still lacking. Another notable gap is the insufficient evaluation of phage activity under normal, non-pathological conditions, as most studies focus on disease-challenge models. Therefore, we plan to conduct a comprehensive meta-review to better elucidate the complex interactions between phages and the gut microbiota, assess their effectiveness across different animal species, and refine dosing strategies to improve therapeutic outcomes.

## Figures and Tables

**Table 1:** Overview of bacteriophage application in pig study under experimental and dietary conditions.

Animals	Phage Administration	Target Bacteria	Remarks	Reference
Young pigs challenged with *Salmonella typhimurium*	Oral—Microencapsulated phage cocktail given via oral gavage (2, 4, 6 h post-challenge)	*Salmonella* *Typhimurium*	No change in fecal samples at 6 h; ileal bacterial loads significantly reduced in phage-treated pigs (1.0 vs. 3.0 log_10_ CFU/mL).	[33]
Finishing pigs	Feed—Phage cocktail	*Salmonella* *Typhimurium*	No significant difference (*p* > 0.05) in *Salmonella* incidence in fecal samples at necropsy.	[34]
Weaning Pigs challenged with *E. coli* K88, K99	Phage cocktail in feed	*E. coli* K88/K99	No significant difference in *E. coli* adherence in ileum/cecum; phages supported gut microbiota composition and reduced inflammation.	[35]
Weanling pigs	1000–1500 mg/kg of phage cocktail in feed	*Salmonella*, *E. coli* (K88, K99, F41), Staph. aureus, *Clostridium perfringens*	Improved ADG, dry matter digestibility, villus height, *Lactobacillus* spp.; reduced coliforms and *Clostridium* spp.	[37]
Intestinal epithelial cells (IECs) of pigs	ETEC K99 and phage EK99P-1 isolated from sewage in a swine farm	*E. coli* K99	Protected intestinal barrier: reduced permeability; preserved tight junction proteins (ZO-1, occludin, claudin-3).	[38]
Growing pigs (Ileal cannulated)	Feed—Phages (0.2%) vs. antibiotics (0.1%)	Phage cocktail (*C. perfringens* types A & C, *E. coli* (f41, k88, and k99), *Salmonella* spp.)	No changes in ileal/fecal nutrient digestibility; both antibiotics and phages improved ileal microbiota composition.	[39]

## Data Availability

No new data were created or analyzed in this study. Data sharing is not applicable to this article.
